# Multi‐country outbreak of Salmonella Stanley ST2045 infections linked to consumption of flavoured noodle products. 1 July 2026

**DOI:** 10.2903/j.efsa.2026.10237

**Published:** 2026-07-07

## Abstract

An outbreak of *Salmonella* Stanley ST2045 is ongoing in the European Union/European Economic Area (EU/EEA), with a total of 106 confirmed cases having been reported by 13 EU/EEA countries (77) and the United Kingdom (29) between November 2025 and June 2026. The outbreak is mainly affecting children and young adults, and has involved at least 49 hospitalisations.

Flavoured noodle products from a specific brand are considered the most likely vehicle of infection in this multi‐country outbreak, with cases reporting having consumed these products in Denmark, Estonia, Germany, Latvia, and Lithuania. The microbiological evidence includes the detection of the outbreak strain in chicken‐ and hot‐chicken noodle products of the same brand in Germany and Lithuania. Traceability investigations linked the products to the same producer in Ukraine as the source. The detection of additional *Salmonella* serovars may suggest the possibility of multiple contamination sources. Control measures have included withdrawals and recalls in several countries, which significantly reduce the likelihood of new *S.* Stanley ST2045 infections related to this outbreak. However, a root cause and point(s) of (cross)contamination have not yet been established, and further investigations are needed. Moreover, given that these products have a long shelf life, they may still pose a risk, as they can be stored in household kitchens for extended periods, meaning that further cases could still occur.

Public health authorities are encouraged to interview any new cases, sequence isolates and share information in EpiPulse. Food safety authorities are encouraged to continue investigating to verify the identified products as the vehicles of infection and to identify the source(s) of (cross)contamination, whether associated with single or multiple ingredients.

Consumers are advised to strictly follow the preparation instructions indicated on labels (if products are not ready‐to‐eat), to ensure proper food hygiene during preparation, and to cook chicken products thoroughly.

Suggested citation: European Centre for Disease Prevention and Control, European Food Safety Authority, 2026. Multi-country outbreak of *Salmonella* Stanley ST2045 infections linked to consumption of flavoured noodle products – 1 July 2026. ISBN: 978-92-9498-905-5, Catalogue number: TQ-01-26-050-EN-N, DOI: 10.2900/8137898.

Also published in EFSA Journal: Scientific report approved by EFSA on 1 July 2026; doi:10.2903/j.efsa.2026.10237 Key words: *Salmonella*, flavoured noodle products, multi-country outbreak, Whole Genome Sequencing (WGS). Requestor: European Commission; Question number: EFSA-Q-2026-00316; correspondence: Ask a question, ISSN: 1831-4732.

## Event background

An outbreak of *Salmonella* Stanley sequence type (ST) 2045 was initially detected by Denmark and reported to the European surveillance portal for infectious diseases (EpiPulse events, 2026-FWD-00022) on 23 March 2026.

After the alert from Denmark, other European countries reported additional cases clustering with the Danish outbreak strain, and this confirmed the multi-country spread of the specific strain of *S.* Stanley. It was observed that children and young adults were disproportionately affected by the outbreak, representing a much larger share of cases than other age groups.

Due to the ongoing risk of infection with *S.* Stanley ST2045 in multiple EU/EEA countries, and a possible common source in the food chain, on 3 June 2026, the European Centre for Disease Prevention and Control (ECDC) and the European Food Safety Authority (EFSA) initiated a joint rapid outbreak assessment to identify the source of the outbreak, assess the risk of infection and trigger public health and food safety investigations.

### Outbreak strain characterisation

The *S.* Stanley outbreak strain was identified as ST2045. As regards antimicrobial resistance markers (analysed using the Resfinder tool), the strain carries only the parC:T57S point mutation, which is not expected to contribute to fluoroquinolone resistance as no additional mutations are present in the quinolone resistance-determining region.

### European outbreak case definition

The European outbreak case definition for a **confirmed outbreak case** is as follows:

A laboratory-confirmed *Salmonella enterica* serovar Stanley case with symptom onset on or after November 2025 (date of sampling or date of receipt by the reference laboratory if date of onset is not available);

AND

fulfilling at least one of the following laboratory criteria for a *S.* Stanley ST2045 isolate sequence:

- clustering in the national cgMLST pipeline within five core genome (cg) allelic differences (alternatively five Single Nucleotide Polymorphisms) from the representative outbreak strain available in EnteroBase (Name: 2602S3177);
- clustering within five cg-allelic differences in single-linkage analysis using ECDC's centralised Molecular Typing Tool;

OR

fulfilling the epidemiological criterion:

- epidemiologically linked to a confirmed case.

## Epidemiological and microbiological investigations of human cases

As of 27 June 2026, a total of 106 confirmed cases of *S.* Stanley ST2045 infections had been reported between November 2025 and June 2026. The isolates from cases belong to a tight genetic cluster and have been identified in Austria (6), Czechia (6), Denmark (10), Estonia (9), France (1), Germany (14), Hungary (1), Latvia (3), Lithuania (23), the Netherlands (1), Norway (1), Poland (1), Sweden (1), and the United Kingdom (29). The overall description of the demographic information is detailed in Table 1.

Furthermore, countries have reported to ECDC that over 30 additional *S.* Stanley cases have been identified since December 2025; however, isolates from these cases have not yet been sequenced.

At least 49 cases have required hospitalisation, indicating severe illness in a substantial proportion of the reported cases. In addition, the outbreak has so far mainly affected children and young adults. The median age was low in all countries reporting more than one case, and 33 cases have been reported in children under 10 years by Austria, Denmark, Germany, Latvia, Lithuania, Norway, and the United Kingdom.

**Table 1. Demographic summary of confirmed *Salmonella* Stanley outbreak cases, stratified by country, in 13 EU/EEA countries and the United Kingdom (n=106 cases), November 2025– June 2026**

| Country | No. of confirmed cases | | | Disease onset (range) | Median age [years] (range) | No. of hospitalised cases |
|---|---|---|---|---|---|---|
| | Total | Female | Male | | | |
| Austria | 6 | 4 | 2 | 4 Feb–22 Mar 2026 | 4.5 (1–33) | 4 |
| Czechia | 6 | 3 | 3 | Nov 2025–Mar 2026 (sample date) | 15 (1–48) | - |
| Denmark | 10 | 1 | 9 | 21 Jan–12 May 2026 | 9 (5–22) | 3 |
| Estonia | 9 | 3 | 6 | 20 Jan–24 May 2026 (sample date) | 10 (1–24) | 8 |
| France | 1 | - | 1 | 8 Feb 2026 | 3 | - |
| Germany | 14 | 6 | 8 | Dec 2025–18 Apr 2026 | 11 (3–26) | 4 (information available for nine cases) |
| Hungary | 1 | 1 | - | 5 May 2026 (sample date) | 6 | - |
| Latvia | 3 | - | 3 | May – June 2026 | 9 (7-11) | 1 |
| Lithuania | 23 | 13 | 10 | 1 Feb–22 May 2026 | 9 (2–20) | 17 |
| Netherlands | 1 | - | 1 | April 2026 | 78 | - |
| Norway | 1 | 1 | - | 6 April 2026 | <10 | - |
| Poland | 1 | 1 | - | 31 Jan 2026 | 26 | 1 |
| Sweden | 1 | - | 1 | 31 May 2026 | 81 | - |
| United Kingdom | 29 | 11 | 18 | 22 Jan–22 May 2026 | 21 (<1–84) | 11 |
| **Total in EU/EEA** | **77** | **33** | **44** | | | **37** |
| **Total in EU/EEA and United Kingdom** | **106** | **44** | **62** | **Nov 2025 – June 2026** | **(1 – 84)** | **49** |

### Information from patient interviews

Interview data on food consumption were reported by eleven countries. No common exposure event or setting was identified across the reporting countries. Only one travel-related case was reported (by Germany in a traveller returning from Thailand).

Epidemiological investigations indicate a link to chicken-flavoured instant noodles and/or processed chicken products. A total of 25 cases had consumed instant noodles: 19 of them had consumed noodles of the same brand (Brand A further linked to the RASFF notification 2026.4131), while two of the 23 cases reported consumption of chicken-flavoured noodles but did not remember which brand. It should be noted that some children had consumed instant noodles without preparation (i.e. uncooked, with seasoning only). Some of the cases that are not yet confirmed as outbreak cases, due to the isolates not yet having been sequenced, also reported consumption of dry instant noodles. Some of these unconfirmed cases also mentioned chicken-flavoured noodles from the same brand (Brand A, as detailed in RASFF 2026.4131).

In addition, there have been recurring reports of chicken consumption in most of the affected countries, with processed chicken products (including nuggets, kebab meat, sausages, and ready-to-eat items) having been frequently consumed. Some cases also reported having consumed unprocessed poultry meat.

In summary, the interview results identified instant noodles and processed chicken products as the most consistent signals from interviews in the countries. Table 2 details the information from patient interviews.

**Table 2. Information from interviews with confirmed cases**

| Country | Number of interviewed cases/total number of cases | Instant noodles | Chicken meat/processed chicken products | Other types of exposure or notes |
|---|---|---|---|---|
| Austria | 5/6 | One case reported consumption of plain, cooked instant noodles from an unknown brand and did not eat the spices included in the package. | All five cases reported consumption of chicken products. An additional interview was conducted with the mother in an affected household comprising three children (aged 1, 2 and 5 years), all three were confirmed as outbreak cases. According to the mother, all three children had eaten pizza topped with chicken kebab meat one day before symptom onset, with the chicken added after baking. The mother also reported developing gastrointestinal symptoms 2–3 days later, though no stool sample was taken. | No contact with pets was reported. |
| Czechia | 6/6 | | Three cases reported consumption of chicken products from different sources, including two different fast-food restaurants. One case had consumed pre-packed chicken (without seasoning). Eggs and other common foods were also mentioned. | One case reported occasional consumption of kebab, as well as pork.<br>With regard to additional food items, apples were reported by three cases, pre-packed cucumber by four cases, and strawberries by two cases.<br>Animal contact was reported for two cases: one case had contact with a pet turtle, and another reported contact with both a dog and a cat. |
| Denmark | 9/10 | Five of the cases with available information reported consuming noodles. Three cases, including the two most recent cases (both children aged <10 years) with onset on 5 and 12 May 2026 had eaten chicken-flavoured noodles from the same brand (Brand A detailed in RASFF 2026.4131). One of the children likes to eat the noodles raw (without adding boiling water), adding the spices but not the oil. | Information on chicken consumption was available for six cases and all six had eaten chicken in the week before symptom onset. However, they had consumed different types of chicken, from different brands, bought in different places. The following processed or ready-to eat chicken products were mentioned in the interviews: frozen kebab chicken meat prepared at home, chicken sausages, roasted chicken bought as take-away, chicken salad, frozen chicken wings, and frozen chicken nuggets. In addition, three cases had consumed chicken breast (bought fresh, not frozen) and two cases had consumed minced chicken meat. | |

| Country | Number of interviewed cases/total number of cases | Instant noodles | Chicken meat/processed chicken products | Other types of exposure or notes |
|---|---|---|---|---|
| Estonia | 3/9 | One case reported eating chicken-flavoured noodles from the same brand (Brand A detailed in RASFF 2026.4131). | | |
| France | 1/1 | | The case reported (and loyalty card confirmed) eating processed chicken products (halal), purchased from a large supermarket chain: cordon bleu (breaded), chicken tenders (breaded) and chicken sausage (cashir au poulet). | Other common food items were mentioned: fresh cucumber, strawberries, raspberries, blueberries, pasteurised cheese, eggs, chocolate egg with toy, and other candies.<br>The case had not consumed instant noodles, travelled or had contact with animals. |
| Germany | 7/14 | Two confirmed cases reported having consumed instant noodle products (one of which was the recalled product (RASFF 2026.4131), the other case could not report which brand of noodles). | Chicken products, including chicken nuggets from fast-food restaurants, were frequently reported among these cases.<br>Four cases reported having eaten chicken nuggets at a fast-food restaurant.<br>Six cases reported having eaten chicken (home-cooked) and boiled or fried eggs. The chicken appears as a signal among the cases and is more frequently reported than in a prior *S.* Bochum outbreak which affected the same age group. | The epidemic curve in Germany suggests prolonged exposure, possibly from a long shelf-life product. No confirmed cases have been reported since the noodles were recalled.<br>Three cases reported having eaten pizza (delivery service). |
| Hungary | 1/1 | The case reported consumption of dry instant chicken-flavoured noodles (from different brands). | The case reported consumption of chicken nuggets at a fast-food restaurant before illness onset. | |
| Latvia | 3/3 | Two cases reported consumption of dry instant noodles before illness onset (Brand A detailed in RASFF 2026.4131). Both cases consumed the noodles uncooked. | | |
| Lithuania | 21/23 | Twelve of the interviewed cases had consumed noodles of the same brand (Brand A detailed in RASFF 2026.4131). Some children consumed the noodles raw. | Chicken consumption (including home-cooked chicken and chicken nuggets) was reported by some cases. | Consumption of eggs was reported by one case, and consumption of pork by another. |
| Sweden | 1/1 | | | The case reported food intake from different Asian-style restaurants/food trucks before falling ill. |
| United Kingdom | 7/29 | One case reported eating chicken-flavoured instant noodles but could not recall the brand. | Most cases reported eating processed chicken (e.g. nuggets) from different retailers. | |

## Microbiological and environmental investigations of food and control measures

This section summarises the results of the food investigations carried out by the food safety authorities in the context of this multi-country outbreak. The traceability and analytical results were shared as of 15 June 2026 by the countries involved under the Rapid Alert System for Food and Feed (RASFF) Alert Notification 2026.4131 (11 European Commission (EC) validated follow-ups, *fups*). Figure 1 displays a graphical representation of the food traceability and microbiological investigations related to the food vehicles suspected in this multi-country outbreak, namely flavoured noodle products. The overall description of the traceability and microbiological information is detailed in Annex 1.

On 11 May 2026 the food safety authority in Germany issued a notification (RASFF 2026.4131) warning that *S.* Stanley had been detected in a sample of chicken-flavoured noodles Product A Brand A Batch A (expiring in April 2027) collected in April 2026 from a retailer. The product was manufactured by the Ukrainian Producer A and imported via the Polish Wholesaler B (2026.4131).

The food safety authority in Lithuania then reported the detection of *S.* Stanley from a hot-chicken flavoured noodle product (Product C Brand A Batch A expiring in April 2027) collected in May 2026 from a retailer, manufactured by the Ukrainian Producer A, and imported via the Polish Wholesaler B. In addition, the authority reported that the product also tested positive for *S.* Richmond and *S.* Newport (*fup7*, 2026.4131).

The food safety authority in Estonia reported that *S.* Stanley had been detected in a beef-flavoured noodle product (Product B Brand A Batch A expiring in April 2027) collected in June 2026 from a retailer, and manufactured by the Ukrainian Producer A (*fup12*, 2026.4131). In addition, the authority reported that a chicken-flavoured noodle product (Product A Brand A from a different batch (Batch B expiring in June 2027)) had tested positive for *S.* Senftenberg (antigenic formula 1,3,19 g,[s],t –). The product was manufactured by the Ukrainian Producer A and traded via the Latvian Wholesaler E (*fup9*, 2026.4131).

The food safety authority in Poland reported that since January 2026, several other noodle products containing many flavourings (chicken, hot-chicken, fried chicken, duck, beef, cheese and bacon, vegetable, shrimp, mix, and curry) had been imported from the Ukrainian Producer A and delivered to EU countries (Lithuania, Germany, Denmark and Czechia), and outside of the EU (United Kingdom) (*fup8*, 2026.4131).

In response to the ongoing national investigations in the EU, the food safety authority in Ukraine, contacted by the European Commission, reported in RASFF that the Ukrainian Producer A had initiated an internal investigation into the cause of the contamination. However, the ingredient used as flavouring (i.e. boiled chicken flavouring) tested negative for *Salmonella* (*fup4*, *fup10, fup11*, 2026.4131).

The food safety authorities of the countries affected by the distribution of the contaminated flavoured noodle products have reported the implementation of control measures (withdrawal from the market and recall from consumers, including the issuing of public warnings).

**Figure 1. Graphical representation of the traceability and microbiological investigations related to flavoured noodle products, as reported by the countries involved to RASFF (as of 15 June 2026)**

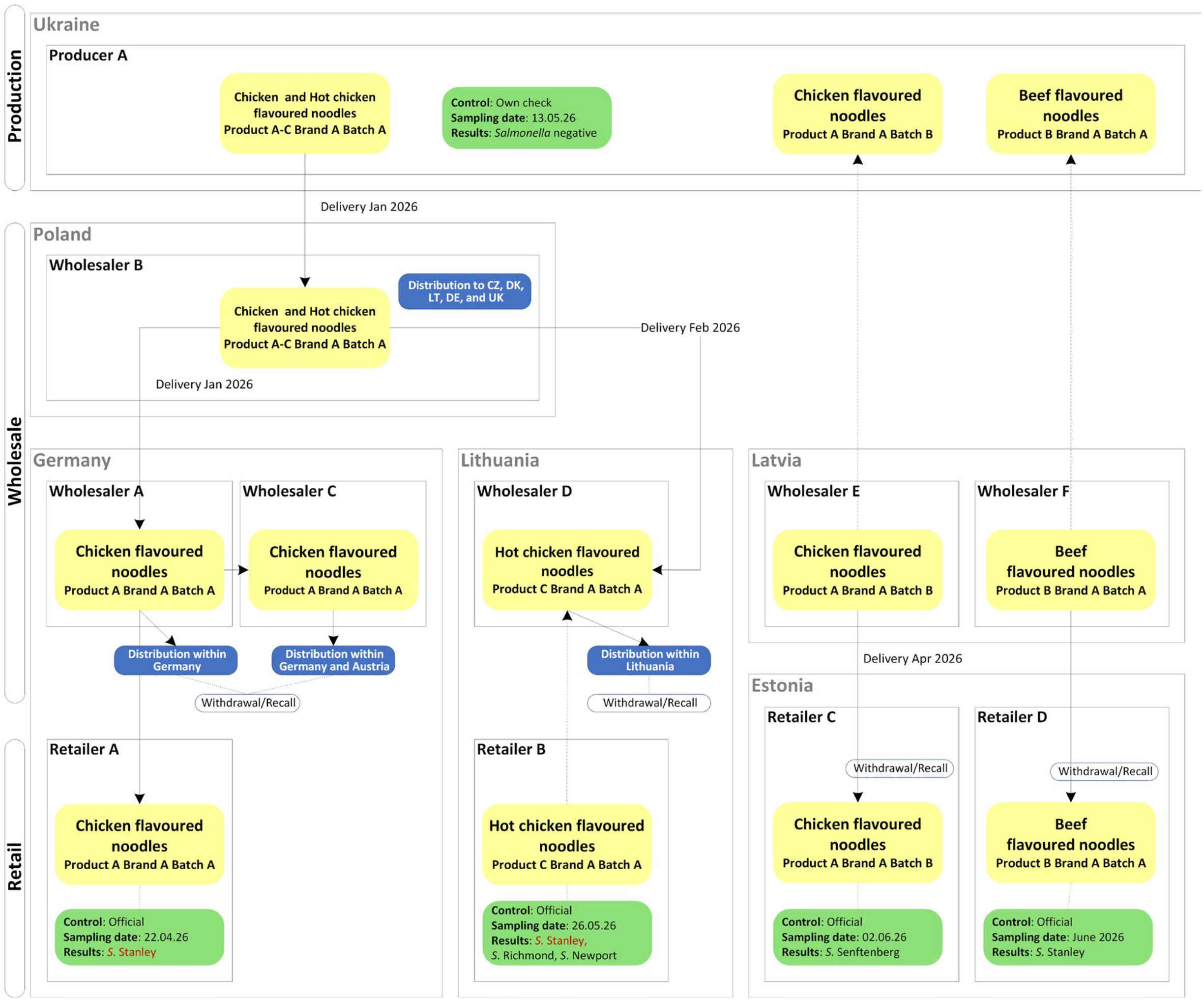

*Green boxes indicate sampling. Red text in green boxes indicates* Salmonella *Stanley findings (outbreak strain). Yellow boxes indicate the products. Blue boxes indicate additional information. Arrows indicate trace back and forward distribution. Boxes refer to affected food business operators and countries.*

## European whole genome sequencing analysis of human and non-human isolates

### WGS data collection and cross-sectoral analysis

#### Human isolates

Cluster analysis was performed on isolate sequences shared in connection with the event and possible matching isolates in ECDC's whole genome sequencing (WGS) database. In total, 49 representative sequences were shared from nine countries: Czechia (6), Denmark (1), Estonia (1), France (1), Lithuania (11), the Netherlands (1), Norway (1), Poland (1) and the United Kingdom (26). All isolates were collected in 2026.

#### Non-human isolates

EFSA launched a call for data in May and in June 2026. Countries were invited to submit genomic information on any collected isolates of *S.* Stanley ST2045 matching the representative outbreak strains to the EFSA WGS System.

#### Cross-sectoral analysis

For cross-sectoral analysis, the cgMLST analysis was performed at both ECDC and EFSA, as previously described [1]. Genome profiles were calculated from assembled genomes using chewBBACA version ≥ 2.8.5, according to the schema described by Rossi et al. 2018 [2] for *Salmonella* enterica, made available by Chewie Nomenclature Server [3]. Isolates with more than 10% of missing loci (325 from a total of 3 255 loci) were excluded from the analysis.

#### Results of cross-sectoral WGS analysis

ECDC queried the EFSA WGS system on 25 June 2026, using the sequence of the representative outbreak strains and European case definition allelic distances as thresholds in the query. In total, two non-human isolates matched the representative human isolate sequences (Figure 2). The two non-human isolates matching the representative outbreak strains are listed below.

- One isolate from chicken-flavoured noodle product (Product A, Brand A, Batch A) collected from the retailer in Germany in April 2026.
- One isolate from hot-chicken-flavoured noodle product (Product C, Brand A, Batch A) collected from the retailer in Lithuania in May 2026.

**Figure 2. Single-linkage cluster tree of 51 human and two non-human *S.* Stanley ST2045 isolate sequences collected in 2026, by country (Czechia, Denmark, Estonia, France, Germany, Lithuania, the Netherlands, Norway, Poland, and the United Kingdom)**

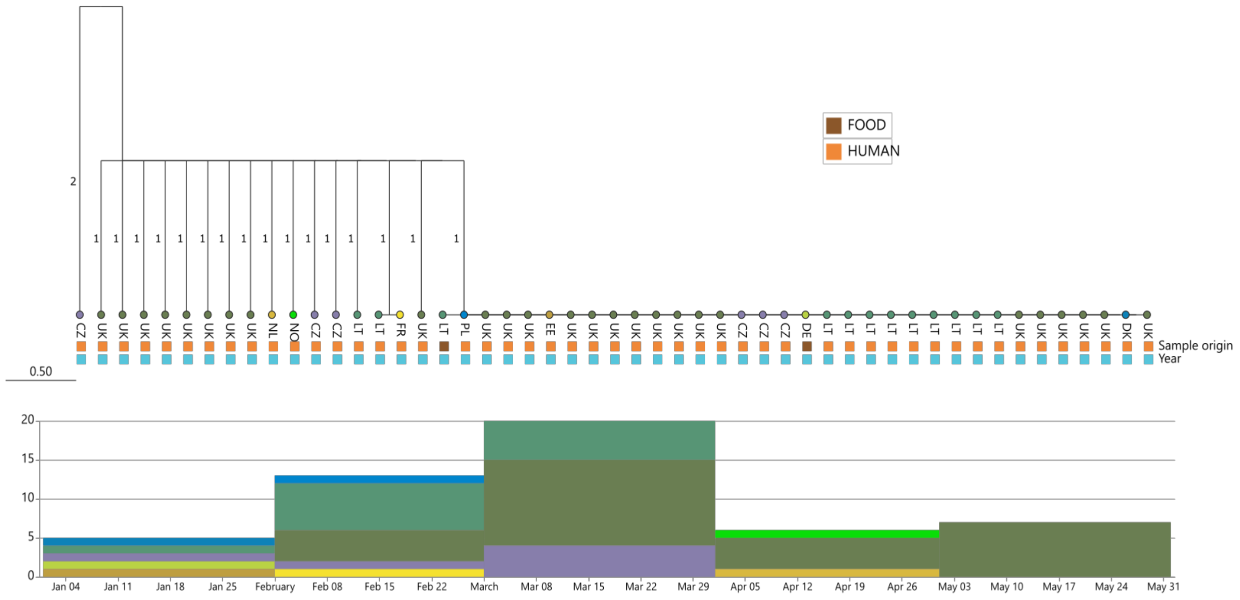

*All isolates are within a 2 AD in a single linkage cluster.*

## ECDC and EFSA risk assessment for the EU/EEA

An outbreak of *S.* Stanley ST2045 is ongoing in the EU/EEA. As of 27 June 2026, 13 EU/EEA countries and the United Kingdom have reported a total of 106 confirmed outbreak cases. The cases were reported between November 2025 and June 2026, with children and young adults disproportionately affected. Reports from the countries indicate a notable public health impact, with at least 49 cases requiring hospitalisation. The countries that have reported cases are Austria (6), Czechia (6), Denmark (10), Estonia (9), France (1), Germany (14), Hungary (1), Latvia (3), Lithuania (23), the Netherlands (1), Norway (1), Poland (1), Sweden (1), and the United Kingdom (29).

Flavoured noodle products from a specific brand are considered the most likely vehicle of infection in this multi-country outbreak, with cases in Denmark, Estonia, Germany, Latvia, and Lithuania reporting having consumed them. However, this exposure does not explain all cases, as some did not report consumption of the implicated products, although most reported consuming noodles and/or processed chicken products. Therefore, additional vehicles of infection cannot be excluded.

The detection of the outbreak strain in chicken and hot-chicken flavoured noodle products from the same brand in Germany and Lithuania further supports the hypothesis of these products as vehicle of infections (as per EU centralised WGS cluster analysis).

The further detection of *S*. Stanley (sequencing ongoing) in Estonia in a beef flavour noodle product of the same brand, traced to the same Ukrainian producer, suggests a common source of contamination at the production plant, potentially linked to either the production environment, or common ingredients used in both productions.

Additional microbiological findings in Lithuania and Estonia related to the detection of other *Salmonella* serovars in chicken-flavoured and hot-chicken flavoured noodle products of the same brand (but different batches), may suggest the possibility of multiple contamination sources. The origin of contamination with multiple serovars should therefore be subject to further investigation to determine whether it is linked to a single or multiple ingredients.

The food safety authorities in the affected countries have reported the implementation of control measures, including withdrawal and recalls, which will reduce the likelihood of new *S*. Stanley ST2045 infections related to this outbreak. However, a root cause and point(s) of (cross)contamination with the outbreak strain have not yet been established. Therefore, further investigations are needed for proper identification and efficient control. In addition, given that these products have a long shelf life, they may still pose a risk, as they can be stored in household kitchens for extended periods, meaning that further cases could occur.

## Recommendations

**Public health authorities** are encouraged to:

- Interview any new cases of *S.* Stanley ST2045, and the other *Salmonella* serovars identified, to investigate potential links to the consumption of instant noodles of different flavours and other products that potentially share ingredients with the instant noodles.
- The infection vehicle identified in this outbreak should be taken into consideration when investigating cases caused by other serotypes: *S.* Senftenberg, *S.* Richmond, and *S.* Newport.
- Sequence human isolates of *S.* Stanley and the other serovars, if possible, and share them in the ECDC WGS system. ECDC can offer support to countries with limited or no sequencing capacity.
- Update the EpiPulse event 2026-FWD-00022, should new cases of *S.* Stanley ST2045 be reported.
- Work closely with food safety authorities on national investigations if new cases of *S.* Stanley ST2045, or cases with the other serovars that may be linked to this outbreak, are identified.

**Food safety authorities** are encouraged to:

- Work with the public health authorities to verify whether the identified products are the vehicle of *S.* Stanley ST2045 infections in the affected EU/EEA countries.
- Continue investigations into the origin of the multiple serovar contamination to determine whether it is linked to a single or multiple ingredients.
- Share in the EFSA WGS system any sequences of the *S.* Stanley ST2045 (and possibly the serovars *S.* Senftenberg, *S.* Richmond, and *S.* Newport), from food and environmental isolates linked to the present event, either microbiologically (serotype or sequence type) or epidemiologically (e.g. suspected food items reported by patients). Share in RASFF the traceability information related to the foods these sequences were derived from.
- Submit genomic data of *S.* Stanley isolates from any kind of food, feed, animal or environmental samples to the EFSA WGS System.

**Consumers** are advised to strictly follow the preparation instructions indicated on the labels when products are not ready-to-eat, and to ensure proper food hygiene during preparation and thorough cooking of poultry meat, including processed chicken products, as inadequate preparation may increase the risk of *Salmonella* infection. Recalled products should not be consumed, but returned to the point of sale or discarded.

## Source and date of request

ECDC sent a request to EFSA on 2 June 2026 to produce a Joint Rapid Outbreak Assessment (ROA). EFSA accepted the request on 3 June 2026.

## Consulted experts and national contact points

**ECDC experts (in alphabetical order):** Cecilia Jernberg, Priyanka Nannapaneni, Elena Portell Buj and Linda Trönnberg.
**EFSA staff (in alphabetical order):** Laura Cabral, Beatriz Guerra, Valentina Rizzi, Mirko Rossi, Eleonora Sarno and Frank Verdonck.

**Public health experts consulted for data and fact validation:**

**Austria**: Sabine Maritschnik, Annika Wolfger and Christian Kornschober (AGES).
**Czechia**: Ondřej Daniel and Michaela Špačková (National Institute of Public Health).
**Denmark**: Stine Nielsen (Department of infectious disease Epidemiology and Prevention, Statens Serum Institut) Gitte Sørensen (Department of Bacteria, Parasites and Fungi, Statens Serum Institut), Luise Müller (Department of infectious disease Epidemiology and Prevention, Statens Serum Institut) and Susanne Schjørring (Department of Bacteria, Parasites and Fungi, Statens Serum Institut).
**Estonia**: Jevgenia Epštein (Chief Specialist, Department of CD Epidemiology, Health Board) and Maria Vikentjeva (Chief Specialist Department of CD Epidemiology, Health Board).
**France**: Alexandra Moura (French National Reference Centre for *E. coli*, *Shigella* & *Salmonella,* World Health Organization (WHO) Collaborating Centre for *Salmonella,* Institut Pasteur, Paris, France) and Genevieve Buser (Division des Maladies Infectieuses, Santé publique France, Saint-Maurice, France).
**Germany**: Anika Meinen (Department for Infectious Disease Epidemiology, Gastrointestinal Infections, Zoonoses and Tropical Infections Unit, Robert Koch Institute); Sophie Hermanns (Department for Infectious Disease Epidemiology, Robert Koch Institute, Berlin, Germany; Postgraduate Training for Applied Epidemiology (PAE), Department for Infectious Disease Epidemiology, Robert Koch Institute, Berlin, Germany; European Programme for Intervention Epidemiology Training (EPIET), European Centre for Disease Prevention and Control, Stockholm, Sweden) and Sandra Simon (Department for Infectious Diseases, NRC for *Salmonella* and other bacterial enteric pathogens, Robert Koch Institute).
**Hungary**: Tünde de Mag (Department of Bacteriology, Mycology and Parasitology Division of Microbiological Reference Laboratories, National Center for Public Health and Pharmacy) and Zsuzsanna Molnár (Department of Communicable Disease Epidemiology and Infection Control, National Center for Public Health and Pharmacy).
**Latvia**: Ilona Čerņakova (Centre for Disease Prevention and Control, SPKC).
**Lithuania**: Jekaterina Sinotova (National Public Health Surveillance Laboratory), Aušra Vailaikienė (National Public Health Service) and Paulius Greičius (National Public Health Surveillance Laboratory).
**Netherlands**: Ninée Buchholtz and Roan Pijnacker (National Institute for Public Health and the Environment, RIVM).
**Norway**: Heidi Lange and Lin Thorstensen Brandal (Department of Infection Control and Preparedness Division of Infectious Disease Control Norwegian Institute of Public Health).
**Poland**: Małgorzata Milczarek-Lisicka and Tomasz Wołkowicz (NIPH NIH – NRI).
**Sweden**: Maarten Coorens and Rikard Dryselius (Public Health Agency of Sweden).
**United Kingdom**: Hannah Charles, Ann Hoban and Gauri Godbole (UKHSA).

**RASFF contact points: Austria, Estonia, Germany, Latvia, Lithuania, Poland**
**Ukraine** (consulted via the European Commission).

**National experts consulted by the RASFF contact points:**
**Austria** Laura Maria Müller (Austrian Agency for Health and Food Safety, AGES).
**Estonia**: Kristi Kallip and Anastasia Rõbakova (Agriculture and Food Board).
**Germany:** RASFF Contact Point team.
**Latvia:** Sarmīte Spiģere and Karina Ruse (Food and Veterinary Service, PDV).
**Lithuania**: Tadas Sakalauskas (State Food and Veterinary Service of the Republic of Lithuania).
**Poland**: RASFF Contact Point team.

**Country Officers of the EFSA WGS system:**
Country Officers of the EFSA WGS System were consulted in Germany and Lithuania.

**National experts consulted by the Country Officers:**
**Germany:** Mathias Ferl (Landesuntersuchungsanstalt für das Gesundheits- und Veterinärwesen Sachsen)
**Lithuania:** Snieguolė Ščeponavičienė and Simona Pilevičienė (National Food and Veterinary Risk Assessment Institute (NMVRVI), Lithuania).

## Disclaimer

This rapid outbreak assessment was written jointly by the European Centre for Disease Prevention and Control (ECDC) and the European Food Safety Authority (EFSA).

ECDC issued this outbreak assessment document in accordance with Article 20 of Regulation (EU) 2022/2371 on serious cross-border threats to health, Articles 7(1) and 8a of Regulation (EC) No 851/2004 establishing a European Centre for Disease Prevention and Control. EFSA's contribution is based on a mandate from the European Commission requesting EFSA to provide scientific assistance from EFSA in the investigation of multinational food-borne outbreaks (Ares (2013) 2576387, Mandate M-2013-0119, 4 July 2013) in accordance with Article 31 of Regulation (EC) No 178/2002 of the European Parliament and of the Council of 28 January 2002, laying down the general principles and requirements of food law, establishing the European Food Safety Authority and laying down procedures in matters of food safety.

The specific purpose of an ECDC-EFSA rapid outbreak assessment is to present an analysis of a cross-border food-borne threat to health and to provide science-based recommendations and options for response. The responsibility for the choice of which options to pursue and which actions to take at national level, following ECDC and EFSA's recommendations, lies with EU/EEA countries.

All data published in this rapid outbreak assessment are data collected from EU and/or EEA countries concerned by the outbreak until the date this assessment was produced. Maps and figures published do not represent statements from ECDC or EFSA on the legal or border status of the countries and territories shown but constitute the information on which this rapid outbreak assessment is based.

## Annex 1. Description of the traceability, food investigations, and control measures by country

### Germany

On 11 May 2026, the food safety authority in Germany issued an RASFF notification (Alert 2026.4131) on the detection of *Salmonella* Stanley in a sample of a composite dish collected as part of an official control on 22 April 2026, from a retailer (German Retailer A). The composite dish consisted of a chicken-flavoured noodle product (Product A Brand A Batch A) expiring in April 2027 (2026.4131).

The German Retailer A received the chicken-flavoured noodle Product A from the German Wholesaler A. The German Wholesaler A had purchased the product in January 2026 from the Polish Wholesaler B, that had bought it from the Ukrainian Producer A in the same month (*fup3*, 2026.4131).

Further investigations revealed that the German Wholesaler A distributed the chicken-flavoured noodle Product A Brand A Batch A to customers within Germany, including the German Wholesaler C that had further distributed it to customers within Germany and Austria.

The food safety authority reported in RASFF that withdrawals and recalls of the batch concerned had been initiated by the German Wholesaler A and the German Wholesaler C, and a consumer notice was issued (*fup1*, 2026.4131).

### Lithuania

On 8 June 2026, the food safety authority in Lithuania reported in RASFF that *Salmonella* Stanley had been detected in a sample of hot-chicken flavoured noodle Product C Brand A Batch A expiring in April 2027. Samples of Product C from the retailer (Retailer B) were tested on 26 May 2026. The authority reported that the product tested positive for *S.* Richmond and *S.* Newport (*fup7*, 2026.4131).

Following the positive findings, the authority carried out an official control at the Lithuanian Wholesaler D, which had distributed the products, and found that there were no longer any products in storage. The wholesaler had received the goods in February 2026 from the Polish Wholesaler B and did not distribute them to other Member States. The authority reported that control measures (withdrawal, recall, and destruction) had been implemented (*fup7*, 2026.4131).

### Austria

On 11 May 2026, the food safety authority in Austria shared in RASFF the outcome of the food investigations it had carried out. The authority reported that the customers receiving chicken-flavoured noodle Product A Brand A Batch A had been contacted, and it also confirmed that the products had been withdrawn from sale and recalled from consumers (*fup2*, 2026.4131).

### Estonia

On 15 June 2026, the food safety authority in Estonia informed RASFF that *Salmonella* Senftenberg (antigenic formula 1,3,19 g,[s],t –) had been detected in a sample of chicken-flavoured noodle Product A Brand A Batch B expiring in June 2027 and manufactured by the Ukrainian Producer A. The product was collected during an official control on 2 June 2026 from the retailer (Retailer C) in response to the RASFF alert. Product A Brand A Batch B was supplied by the Latvian Wholesaler E. Control measures consisted of a public recall sent to retailers on 10 June 2026. The products were withdrawn and removed from the market (*fup9*, 2026.4131).

Furthermore, the authority reported the detection of *Salmonella* Stanley from a beef-flavoured noodle product (Product B Brand A Batch A expiring in April 2027) collected during an official control (June 2026) from the retailer (Retailer D), and manufactured by the Ukrainian Producer A (*fup12*, 2026.4131). Product B was supplied by the Lavian Wholesaler F. The products were withdrawn and removed from the market (*fup12*, 2026.4131).

### Poland

On 27 May 2026, the food safety authority in Poland reported in RASFF that an official control had been carried out at the Polish Wholesaler B. During the control, the authority found out that the Polish Wholesaler B had purchased the goods (chicken-flavoured noodle Product A Brand A Batch A) in January 2026 from the Ukrainian Producer A (*fup4*, 2026.4131). The authority also reported in RASFF that the Polish Wholesaler B had informed the Ukrainian Producer A of the *Salmonella* detection in Batch A. In reply, the Ukrainian Producer A explained that it had initiated an internal investigation into the cause of the contamination. Furthermore, according to the laboratory report received by the Polish Wholesaler B, analysed samples of chicken-flavoured noodle Product A Brand A Batch A (in storage at the Ukrainian Producer A) tested negative for *Salmonella* on 13 May 2026 (*fup4*, 2026.4131).

The authority also reported that the Polish Wholesaler B had imported several noodle products from the Ukrainian Producer A since January 2026 and that these had contained many flavourings (chicken, hot-chicken, fried chicken, duck, beef, cheese and bacon, vegetable, shrimp, mix, and curry). These products were delivered to EU countries (Lithuania, Germany, Denmark, and Czechia), and outside of the EU (United Kingdom) (*fup8*, 2026.4131).

### Ukraine

On 15 June 2026, the food safety authority in Ukraine, contacted by the European Commission, reported the results of the investigations carried at the Ukrainian Producer A in RASFF. The authority reported that an official control had been performed and that the ingredient used at the plant as flavouring (i.e. boiled chicken flavouring) had been analysed and had tested negative for *Salmonella*. The authority also clarified that the flavoured noodle products are a semi-prepared food, requiring cooking before consumption (i.e. not ready-to-eat), as specified by the manufacturer's instructions on the label (*fup4*, *fup10, fup11*, 2026.4131).

## Annex 2. Food-borne outbreaks caused by *S*. Stanley

Country specific data on food-borne outbreaks associated with *S*. Stanley are reported to EFSA by countries in accordance with the Zoonoses Directive 2003/99/EC. Overall, six outbreaks were reported between 2020 and 2024. The two strong-evidence foodborne outbreaks have been reported by one EU Member States: Italy (two outbreaks in 2023), with overall 94 human cases, 43 hospitalisations, and no deaths. The reported food vehicles were 'Buffet meals' and 'Pig meat and products thereof'. The four weak-evidence foodborne outbreaks have been reported by three EU Member States: France (two outbreaks in 2020 and 2022), Slovenia (2021), and Italy (2023), with 10 human cases overall, three hospitalisations, and no deaths. The reported food vehicles were 'Eggs and egg products', 'Mixed food', 'Unknown', and 'Pig meat and products thereof'.
